# Molecular Evolution and Expression Analysis of the ADH Gene Family in Apple Bud Mutants

**DOI:** 10.3390/biology15171486

**Published:** 2026-09-02

**Authors:** Shuai Ye, Miao Shao, Mingyu Chu, Baihong Chen, Juan Mao

**Affiliations:** State Key Laboratory of Aridland Crop Science, Co-Built by the Provincial and Ministerial Authorities, College of Horticulture, Gansu Agricultural University, Lanzhou 730070, China; 18750911869@163.com (S.Y.); 15842120776@163.com (M.S.); chumy@gsau.edu.cn (M.C.); bhch@gsau.edu.cn (B.C.)

**Keywords:** apple, ADH gene family, bud mutant, genome-wide characterization, molecular evolution

## Abstract

Alcohol dehydrogenase (ADH) catalyzes the reduction of aldehydes to alcohols, key precursor substrates for volatile ester biosynthesis, which determines the characteristic aroma of apple fruit. However, a comprehensive genome-wide investigation of the ADH gene family in apple has been lacking. In this study, we systematically identified ADH genes in the apple genome using integrated bioinformatics approaches, including phylogenetic analysis, synteny evaluation, promoter cis-element prediction, codon usage bias assessment, and protein interaction network modeling. Expression patterns were examined through transcriptomic data and validated by RT-qPCR analysis across different organs and among ‘Red Delicious’ and its four bud mutant lines. We identified 44 *ADH* genes, with 12 forming a prominent cluster on chromosome 1. RT-qPCR analysis revealed that *MdADH20* was dramatically upregulated in the ‘Red Chief’ mutant (relative expression of 59.38), suggesting its pivotal role. Phylogenetic analysis revealed a close evolutionary relationship with wild strawberry. The encoded proteins were generally stable and predominantly localized to the cytoplasm. Promoter analysis showed enrichment of growth/development-related and ARE elements, while codon usage analysis identified AGA, GCU, GUU, and CUU as preferred codons. Protein interaction prediction suggested MdADH19 and MdADH20 as hub proteins. Expression profiling and RT-qPCR further identified *MdADH20* as a core candidate gene, characterized by its stable and high expression, particularly in the ‘Red Delicious’ mutant. Its central position in the predicted protein–protein interaction network suggests a potential regulatory role in the aroma biosynthesis pathway of apple fruit. This study provides the first systematic genome-wide characterization of the apple ADH gene family, establishing a theoretical groundwork for deciphering aroma biosynthesis mechanisms and offering potential target genes for flavor improvement through bud mutation breeding strategies.

## Simple Summary

The appealing scent of an apple is one of the main reasons consumers choose it, and this fragrance comes mainly from a group of compounds called volatile esters. Their production depends on several enzymes, among which alcohol dehydrogenase (ADH) plays an indispensable role by providing the raw materials for ester synthesis. In this study, we scanned the entire apple genome and identified 44 genes belonging to the ADH family. Notably, 12 of these genes are located close together on a single chromosome, suggesting they may have arisen through gene duplication during evolution. By examining gene activity in different plant parts and comparing the original ‘Red Delicious’ variety with four of its bud-derived mutants, we found that one particular gene, *MdADH20*, was extremely active in the ‘Red Chief’ mutant, making it a strong candidate for involvement in aroma production. Protein interaction predictions further pointed to MdADH19 and MdADH20 as central players in the regulatory network. This study offers the first comprehensive view of the ADH gene family in apple and provides promising targets for future breeding efforts aimed at enhancing fruit flavor.

## 1. Introduction

As a prominent temperate fruit tree belonging to the Rosaceae family, apple (*Malus domestica*) occupies a pivotal position in the global fruit industry. Fruit quality directly influences consumer sensory perception and commercial value, among which aroma profiles are particularly critical for enhancing market appeal. The characteristic scent of apple fruit is mainly derived from volatile esters [1,2], which constitute the core elements that shape its distinctive sensory attributes.

The biosynthesis and accumulation of aroma volatiles are governed by intricate genetic regulation and environmental factors, representing a highly complex metabolic network. The lipoxygenase (LOX) pathway serves as the principal route for generating precursor compounds of characteristic apple aromas. Within this regulatory framework, alcohol dehydrogenase (ADH) functions as a crucial rate-limiting enzyme, catalyzing the reductive conversion of aldehydes into alcohols [3,4]. ADH activity directly modulates the metabolic flux through the LOX pathway [3], thereby playing a decisive role in the ultimate accumulation of aroma volatiles in fruit. Moreover, the alcohols produced via ADH catalysis not only act as direct aroma contributors but also serve as essential substrates for ester biosynthesis [5]. Consequently, the ADH gene family occupies a central regulatory position in the accumulation of alcohols and esters in fruit, with its expression and functional status intimately linked to apple aroma quality. Nevertheless, genome-wide identification and phylogenetic appraisal of the ADH gene family in apple have remained unreported until now.

In horticulture, gene families play critical roles in plant metabolism. Genome-wide identification is typically the first step in studying a given family, aiming to retrieve all members from the target genome [6,7,8]. This is generally achieved through two complementary approaches: homology-based searching, which uses known protein sequences from model species as queries [9,10], and domain-based searching, which employs hidden Markov models of conserved domains to detect candidate genes regardless of overall sequence similarity [11,12]. Following identification, functional characterization of individual members is conducted to elucidate the regulatory networks governing plant metabolic processes [13,14,15]. Considerable progress has been achieved in ADH gene family studies in model species and horticultural crops, such as Arabidopsis (*Arabidopsis thaliana*) [16], tomato (*Solanum lycopersicum* L.) [17], and wild strawberry (*Fragaria vesca* L.) [18]. For instance, the numbers of *ADH* genes identified in rapeseed [19], tobacco [20], tomato [17], and grape genomes [21] are 47, 53, 35, and 11, respectively. Within Rosaceae, investigations into *ADH* genes in pear (*Pyrus* L.) and apple have also revealed complex orthologous and paralogous relationships [22,23]. Additionally, the apple genome has undergone multiple rounds of whole-genome duplication [24,25], driving substantial expansion and functional divergence of gene families. This evolutionary history provides a genetic basis for aroma diversity but concomitantly increases the complexity of gene family analysis. Although considerable knowledge has been accumulated regarding *ADH* genes in other species, a systematic genomic characterization of the ADH gene family specifically within the apple LOX pathway remains lacking.

Additionally, some progress has been made in understanding the regulatory roles of *ADH* genes in apple volatile biosynthesis. For example, Jamil et al. reported that suppression of *ADH* gene expression via ethylene spraying or bagging treatments led to reduced overall aroma synthesis, decreased ADH enzyme activity, aldehyde accumulation, and diminished total ester content [26]. Feng et al. observed that concurrent upregulation of *MdLOX*, *MdAAT2*, and *MdADH3* resulted in a notable increase in ester content [27]. Beyond interspecific comparisons, exploring the molecular mechanisms underlying aroma variation using mutant lines with identical genetic backgrounds offers a unique vantage point for dissecting *ADH* gene functions. However, most previous studies have relied on comparisons across different cultivars or treatments, where genetic background differences may confound direct assessments of *ADH* function. Bud mutants arise from somatic mutations during asexual propagation, which can induce heritable changes in fruit traits that are transmitted to progeny, and have thus become a valuable resource for apple breeding [28,29]. Unlike conventional cultivar comparisons, which are confounded by extensive genetic background variation, bud sports are isogenic lines that differ only by spontaneous somatic mutations. This unique genetic uniformity provides an ideal system to identify genes responsible for phenotypic variation, such as altered aroma profiles, and to validate candidate genes discovered through genomic analyses. Nevertheless, research on the impact of bud mutations on fruit aroma is relatively scarce [28,29,30,31], with the majority of studies focusing on aroma component differences among apple varieties from distinct geographical origins or growth conditions. Bud mutant materials offer an ideal genetic context for validating candidate genes identified through genomic screening. Therefore, elucidating the molecular mechanisms by which bud mutations alter fruit aroma in apple is a worthwhile research pursuit.

A thorough understanding of the genomic features and expression regulation patterns of the ADH gene family is a prerequisite for deciphering the molecular mechanisms through which the apple LOX pathway governs aroma synthesis [3,4,23]. Such knowledge will also provide important genetic resources and a theoretical foundation for the targeted enhancement of apple flavor quality via genetic engineering. However, a systematic genome-wide investigation of the ADH gene family in apple, particularly its expression regulation in bud sport materials, has remained elusive. Accordingly, this study conducts a systematic genome-wide identification and comprehensive analysis of the ADH gene family in apple, focusing on its evolutionary history, functional attributes, and expression profiles, and codon usage bias analysis, which can provide insights into the evolutionary forces shaping gene sequences and offer references for heterologous expression [32]. By employing structural diversity, and expression profiles across different tissues and among ‘Red Delicious’ and its multi-generation bud mutant derivatives via RT-qPCR screening for core candidate genes [28,29], this research aims to advance our understanding of the molecular contributions of *ADH* genes to apple fruit aroma formation and to provide scientific support for breeding apples with superior flavor qualities.

## 2. Materials and Methods

### 2.1. Plant Materials

In this investigation, mature ‘Red Delicious’ apples (140 days after full bloom) were collected from the Tianshui Apple Demonstration Orchard in China. Additionally, four successive generations of bud sport mutants: ‘Starking Red’, ‘Starkrimson’, ‘Red Chief’, and ‘Oregon Spur II’ were included. These five genotypes, designated as Y, L, X, S, and E according to their mutation generations, were systematically categorized. To ensure experimental consistency, all samples were harvested at the same developmental stage, with peel tissues from three individual fruits per genotype serving as biological replicates. At the time of harvest, the average diurnal temperature was 25 ± 3 °C, with a relative humidity of 65–70%. The collected peel samples were immediately snap-frozen in liquid nitrogen to prevent degradation and stored at −80 °C until subsequent RT-qPCR analysis and gene expression assessment.

### 2.2. Systematic Characterization of the ADH Gene Family Across the Apple Genome

To comprehensively identify the ADH gene family within apples, genomic sequences of *Malus domestica* and ADH protein sequences from *Arabidopsis thaliana* were retrieved from the Phytozome database (http://www.phytozome.net, accessed on 15 October 2025) to be used as search references [33], the apple reference genome v3.0) from the Phytozome database. Homology searches were conducted with HMMER software (v3.3.2) [34], utilizing hidden Markov models relevant to the conserved ADH domains PF00107 and PF08240. The candidate protein sequences identified were then analyzed through the Pfam (http://pfam.xfam.org/, accessed on 15 October 2025) [35], CDD (https://www.ncbi.nlm.nih.gov/cdd/, accessed on 15 October 2025) [36], and ProSite (http://prosite.expasy.org/, accessed on 15 October 2025) databases for domain validation, successfully confirming that all candidates had both the ADH_N and ADH zinc N conserved motifs.

### 2.3. Phylogenetic Reconstruction and Synteny Analysis of ADH Genes

Using ClustalX2 (version 2.1) [37], multiple sequence alignments of apple ADH proteins were generated alongside their orthologs from plum (*Prunus mume* Siebold & Zucc.), wild strawberry (*Fragaria vesca* L.), and rose (*Rosa chinensis* Jacq.). Phylogenetic relationships were inferred using the Neighbor-Joining algorithm within the MEGA 12 framework [38], based on sequence alignment data. Branch robustness was evaluated through 1000 bootstrap iterations, and the final tree topology was rendered using the iTOL web platform (https://itol.embl.de, accessed on 18 October 2025) [39]. For collinearity analysis, both intra-genomic (within apple) and inter-genomic (between apple and the other three species) syntenic relationships were examined and illustrated using TBtools-II software (https://github.com/CJ-Chen/TBtools, accessed on 18 October 2025) [40], which was also used to generate the corresponding collinearity maps.

### 2.4. Physicochemical Characterization of ADH Proteins

Chromosomal positions of apple *ADH* genes were obtained from the Phytozome repository [41], and their physical distribution along chromosomes was visualized using TBtools-II. For each deduced protein, a suite of physicochemical parameters—namely amino acid length, molecular weight, theoretical pI, instability index, aliphatic index, and GRAVY—was calculated via the ExPASy ProtParam portal (https://web.expasy.org/protparam/, accessed on 24 October 2025) [39]. Additionally, the subcellular compartment where each protein is likely to localize was predicted through WoLF PSORT (https://wolfpsort.hgc.jp/, accessed on 24 October 2025) [42].

### 2.5. Analysis of Gene Architecture and Conserved Sequence Elements

Extraction of exon–intron structural information for apple *ADH* genes was performed based on the GFF annotation file of the reference genome, with graphical representation of gene architectures produced via TBtools-II. Identification of conserved peptide motifs was carried out through the MEME suite (http://meme-suite.org/tools/meme, accessed on 24 October 2025) [41], applying criteria that allowed for up to ten motifs per query, each spanning 6–50 amino acids. Display of the identified motif arrangements along gene sequences was subsequently achieved with TBtools-II [40].

### 2.6. Codon Usage Bias Analysis

From the apple ADH gene family, we obtained the coding sequences (CDS) and subsequently employed CodonW (v1.4.2) to compute multiple parameters reflective of codon usage bias, namely the effective number of codons (ENC), codon adaptation index (CAI), codon bias index (CBI), and relative synonymous codon usage (RSCU) [43]. Those codons exhibiting RSCU values exceeding 1.0 were designated as preferentially used. Moreover, we explored the association between GC composition at the wobble position (GC3s) and the ENC estimates.

### 2.7. Promoter Cis-Element Profiling and Functional Dissection

Retrieval of the 2000 bp sequences flanking the start codons of apple *ADH* genes was performed, followed by their submission to the PlantCARE webserver (http://bioinformatics.psb.ugent.be/webtools/plantcare/html/, accessed on 26 October 2025) for cis-element scanning. Based on their functional annotations, the identified motifs were grouped into four broad categories—light-responsive, stress-responsive, hormone-responsive, and development-associated. TBtools-II served as the visualization tool for mapping these elements onto the promoter regions [40].

### 2.8. Tissue-Specific Expression Profiling

Of the 44 identified *ADH* genes, we focused our study on 20 of them, which form distinct evolutionary clades with close phylogenetic relationships; therefore, we conducted tissue-specific analyses on these 20 genes. FPKM-based transcript abundance of apple *ADH* members across roots, stems, leaves, flowers, and fruits was sourced from the AppleMDO database (http://bioinformatics.cau.edu.cn/AppleMDO/, accessed on 28 October 2025) [44] To illustrate the expression landscape, we employed TBtools-II for heatmap construction, and subsequently applied hierarchical clustering to detect tissue-dependent expression behaviors [40].

### 2.9. Protein–Protein Interaction Network Prediction

The apple ADH protein sequences were submitted to the STRING database (https://string-db.org/) with the organism set to apple and the minimum required interaction confidence score set at 0.900 [45], To ensure the high reliability of the predicted interactions, we set the minimum required interaction confidence score to 0.900, which is the ‘highest confidence’ threshold recommended by STRING database for stringent analyses. The resulting TSV interaction output was then imported into Cytoscape software (v3.9.1) for network visualization, where node sizes were adjusted based on betweenness centrality values [46].

### 2.10. Quantitative Real-Time PCR (RT-qPCR) Analysis

From the 44 identified ADH genes, we selected 20 genes that formed independent clades with close phylogenetic relationships to serve as our study material. Each variety of each gene was tested in triplicate. Gene-specific oligonucleotide primers for RT-qPCR analysis were designed using Primer Premier 5 based on the ADH sequences we had identified, with all primer pairs listed in Table 1. Synthesis was commissioned to Geneworks Biotechnology Co., Ltd. (Shanghai, China), and the apple *Actin* gene was chosen as the endogenous control. Thermal cycling and fluorescence signal acquisition were performed on the LightCycler^®^ 96 Real-Time PCR System (Roche, Basel, Switzerland), and relative expression levels were determined using the 2^−ΔΔCT^ calculation method under the following conditions: an initial denaturation at 95 °C for 30 s, followed by 40 cycles, each consisting of denaturation at 95 °C for 5 s and annealing/extension at 60 °C for 30 s. Melting curve analysis was carried out by heating from 60 °C to 95 °C to verify the specificity of amplification. All reactions were performed in a total volume of 10 μL with a hot lid temperature of 100 °C, each 10-μL reaction system comprised 3 μL of ddH_2_O, 1 μL of cDNA, 0.5 μL of each primer, and 5 μL of SYBR Green Master Mix. FastStart Essential DNA Green Master (Roche Diagnostics Ltd., Rotkreuz, Switzerland) was used for RT-qPCR amplification on a LightCycler^®^ 96 Real-Time PCR System (Roche Diagnostics Ltd., Rotkreuz, Switzerland).

### 2.11. Statistical Treatment and Data Analysis

Statistical evaluation of the experimental data was conducted using Excel 2021 and IBM SPSS Statistics 25. To determine whether gene expression levels differed significantly, we performed one-way analysis of variance (ANOVA) coupled with Duncan’s multiple range test at a significance threshold of *p* < 0.05. All figures were produced with Origin 2021 [47].

## 3. Results

### 3.1. Identification and Chromosome Distribution Analysis of Apple ADH Genes

**Figure 1:**
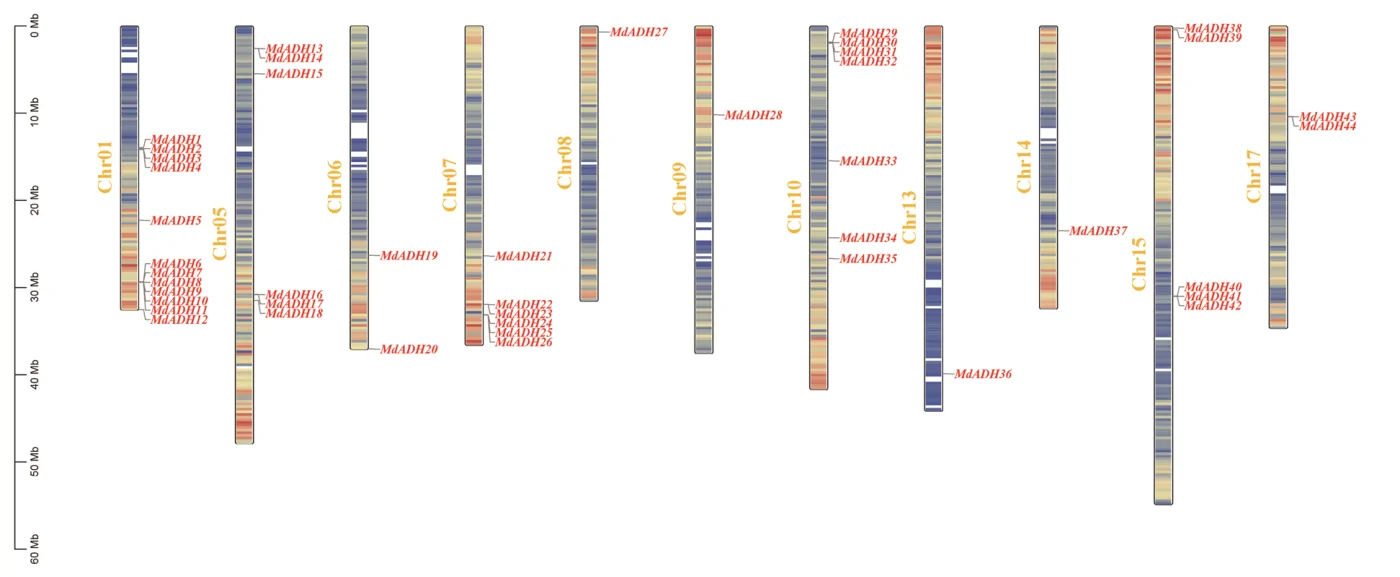
Chromosomal positions of *MdADH* genes were obtained from the Phytozome database and visualized using TBtools-II. Chromosomal distribution of ADH family members in the apple (*Malus domestica*) genome. The *ADH* genes are mapped onto their respective chromosomes. The scale bar on the left indicates chromosome length in megabases (Mb).

A genome-wide survey of the apple genome uncovered 44 full-length genes harboring both ADH_N and ADH_zinc_N domains, which were systematically named *MdADH1* through *MdADH44*. These loci were found to be unevenly dispersed across 14 chromosomes, with a notable aggregation of 12 members (*MdADH1*–*MdADH12*) on Chr01. The distribution of *ADH* genes on other chromosomes is more scattered, with Chr05, Chr07, Chr10, and Chr15 each containing 4–6 genes, while no *ADH* genes were detected on chromosomes such as Chr02, Chr03, Chr04, Chr11, Chr12, and Chr16 (Figure 1). Beyond the overall distribution pattern, we noted that the 12 genes on Chr01 were arranged in three distinct subclusters: a proximal cluster, a middle cluster, and a distal cluster. This discontinuous clustering pattern suggests that the Chr01 locus has undergone multiple independent tandem duplication events rather than a single large-scale duplication. Additionally, Chr05 contained six members distributed across two separate regions, while the five members on Chr07 were all confined to a single contiguous block, representing the most compact cluster in the genome.

### 3.2. Physicochemical Properties and Subcellular Localization of Proteins

Characterization of the 44 ADH proteins with respect to their physicochemical parameters showed substantial variation in chain length, spanning from 107 aa (MdADH28) to 415 aa (MdADH17), with a mean of 366 aa. Corresponding molecular masses fell between 12.12 and 60.99 kDa. The theoretical pI values ranged from 5.43 to 9.49, with acidic (pI < 7) and basic (pI > 7) isoforms each constituting roughly half of the family. Instability assessment revealed that only MdADH38 exhibited a coefficient exceeding 40 (40.62), accounting for 2.27% of all members. The aliphatic index varied from 73.60 to 102.05, while the GRAVY scores ranged between −0.396 and 0.111, with the vast majority (93.18%) showing negative values. Subcellular localization predictions further indicated that most ADH proteins (approximately 70.5%) were destined for the cytoplasm, with the remainder distributed among the cytoskeleton, the extracellular milieu, or the chloroplasts (Table 1). Correlation analysis between amino acid length and subcellular localization revealed that the four proteins targeted to non-cytoplasmic compartments (MdADH28 to mitochondria, 107 aa; MdADH27 and MdADH39 to peroxisomes, 370 and 366 aa, respectively; MdADH38 to chloroplast, 391 aa) exhibited an average length of 308.5 aa, notably shorter than the cytoplasmic group average of 382.6 aa. This length difference implies that N-terminal or C-terminal extensions may be required for cytoplasmic retention, and their loss might facilitate organellar targeting. Furthermore, the sole unstable protein (MdADH38, instability index = 40.62) was also the only chloroplast-targeted member, suggesting that its metabolic environment may demand distinct structural properties.

### 3.3. Phylogenetic Analysis

**Figure 2:**
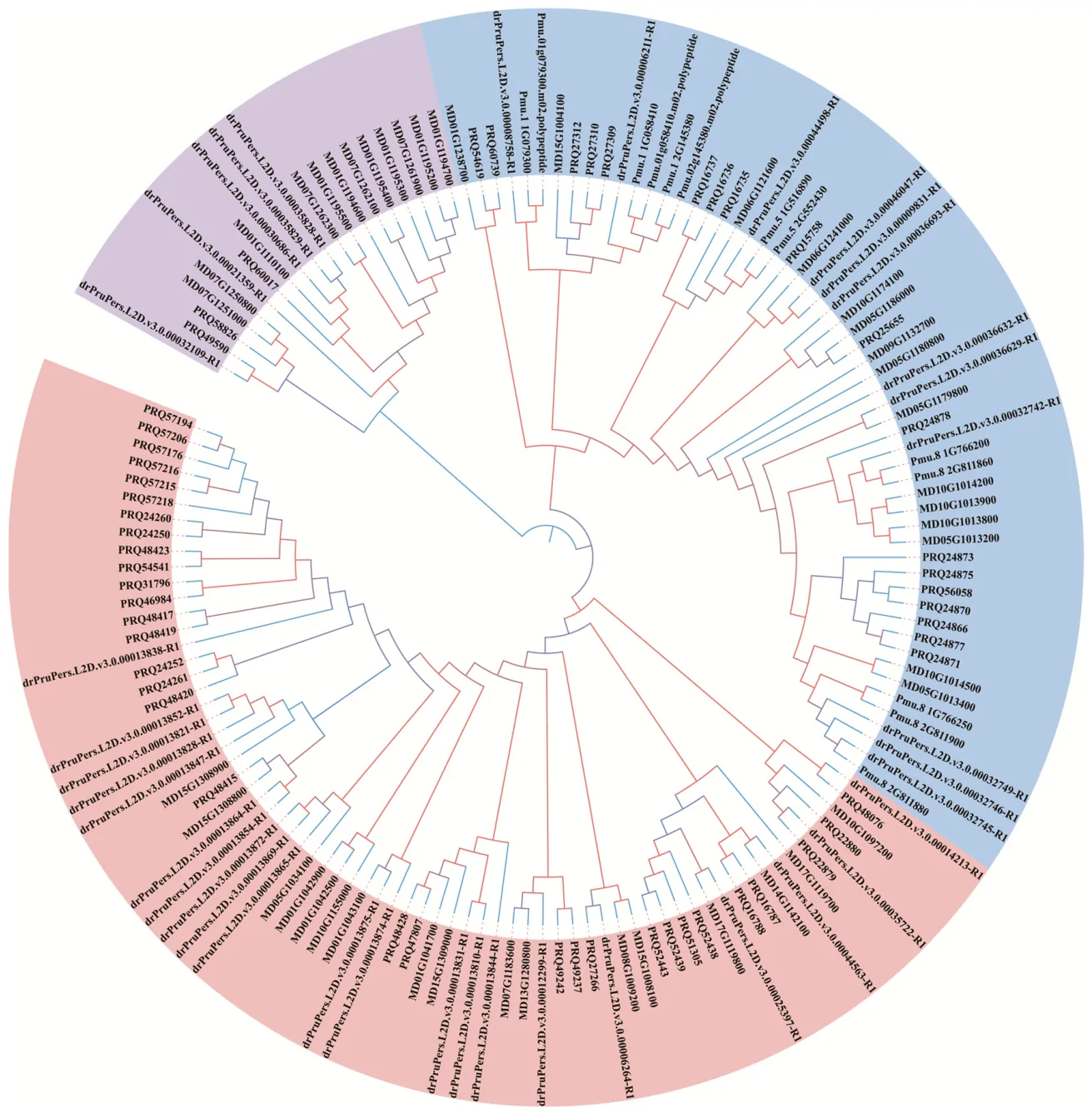
Interspecific phylogenetic tree of ADH proteins from apple (Md: *Malus domestica*), plum (Pm: *Prunus mume* Siebold & Zucc.), wild strawberry (Fv: *Fragaria vesca* L.), and rose (Rc: *Rosa chinensis* Jacq.). The phylogenetic tree was constructed using the Neighbor-Joining (NJ) method with 1000 bootstrap replicates in MEGA 12. Different colors indicate clustering patterns. The gene identifiers for apple shown in the figure are prefixed with ‘MD’ and follow the same naming convention as the gene IDs in Table 1. Refer to Table 1 to look up the corresponding gene names based on the gene IDs.

We aimed to resolve the evolutionary affinities among apple *ADH* genes by aligning the 44 apple protein sequences against homologous *ADH* members from plum (76), wild strawberry (19), and rose (47). Following the alignment, we reconstructed a phylogenetic tree to infer their genealogical relationships (Figure 2). The resulting phylogeny indicated that the ADH proteins from these four Rosaceae species could be divided into three distinct subfamilies, consisting of 20, 58, and 70 members, respectively. Apple *ADH* genes are distributed across all three subfamilies. Among them, the *ADH* genes of apple and wild strawberry are evolutionarily closely related, with their members clustering together in multiple branches. Apple is relatively distantly related to plum and rose, but some genes still exhibit cross-species clustering patterns. Within the three subfamilies recovered from the phylogeny, the distribution of apple ADH proteins was as follows: Subfamily I contained 6 apple members (13.6% of total), Subfamily II contained 22 members (50.0%), and Subfamily III contained 16 members (36.4%). Notably, all six members of Subfamily I were derived from the Chr01 tandem cluster, whereas Subfamily II and III members were distributed across multiple chromosomes. This non-random association between phylogenetic clade and chromosomal origin suggests that the Chr01 cluster may represent an evolutionarily ancient subfamily that has undergone extensive local expansion, while the other subfamilies arose from more dispersed ancestral copies.

### 3.4. Analysis of Conserved Motifs and Gene Structure

**Figure 3:**
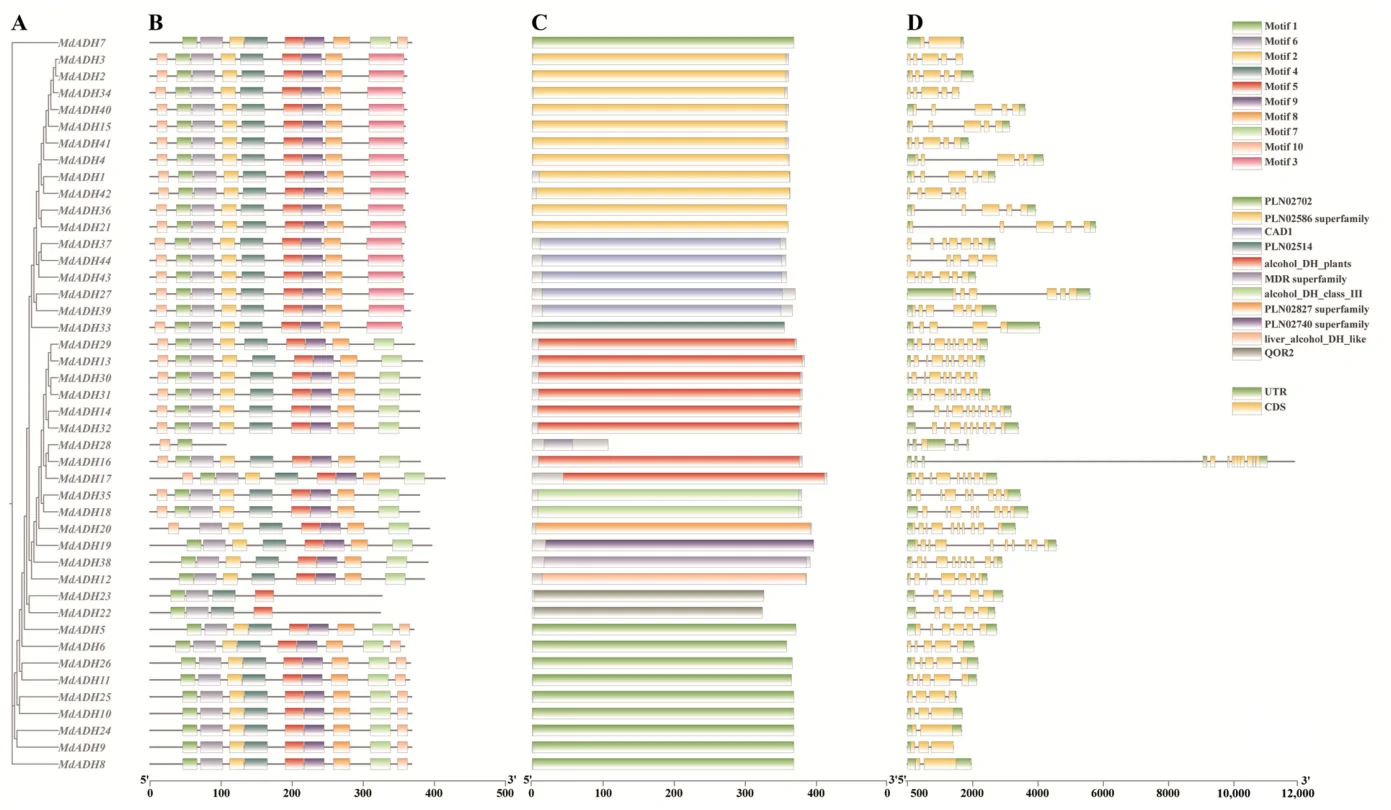
Phylogenetic relationships, conserved motifs, conserved domains, and intron–exon structures of MdADH gene family. Conserved motifs were identified using MEME. (**A**) Phylogenetic relationships of MdADH proteins. (**B**) Distribution of ten conserved motifs (Motif 1-10). (**C**) Conserved functional domains of MdADH proteins. (**D**) Intron–exon structure of *MdADH* genes.

Application of the MEME tool to the apple ADH protein set resulted in the detection of ten conserved motifs (designated Motif 1 through Motif 10) (Figure 3). Inspection of their distribution revealed that proteins within the same subfamily generally shared analogous motif patterns. A core set of seven motifs (Motif 1, 2, 4, 5, 6, 8, and 9) was found to be conserved across all ADH members, whereas certain proteins—MdADH16, for instance—carried unique motif combinations absent from other family members. Subsequent domain annotation confirmed the presence of the alcohol_DH_plants conserved domain in every ADH protein, corroborating their identity as plant ADH enzymes. In addition, several members harbored auxiliary domains, including QOR2 and CAD1. With regard to gene structure, the exon number among apple *ADH* genes varied from 2 to 10, with the vast majority containing five exons. Notably, gene architectures were largely conserved within each subfamily, whereas clear inter-subfamily differences were observed, mainly manifested as variations in intron lengths and exon counts. Motif 3, which was absent from the majority of apple ADH proteins, was detected exclusively in the five members of Subfamily III that also harbored the auxiliary CAD1 domain (*MdADH16*, *MdADH17*, *MdADH18*, *MdADH40*, and *MdADH41*). This perfect co-occurrence (5/5, 100%) between Motif 3 and the CAD1 domain implies that this motif may be functionally linked to cinnamyl alcohol dehydrogenase-related activities. Conversely, Motif 7 and Motif 10 were both present in all Subfamily I members but were completely absent from the other two subfamilies, suggesting that these two motifs may serve as diagnostic signatures for the derived subclade of Chr01.

### 3.5. Promoter Cis-Regulatory Element Profiling

**Figure 4:**
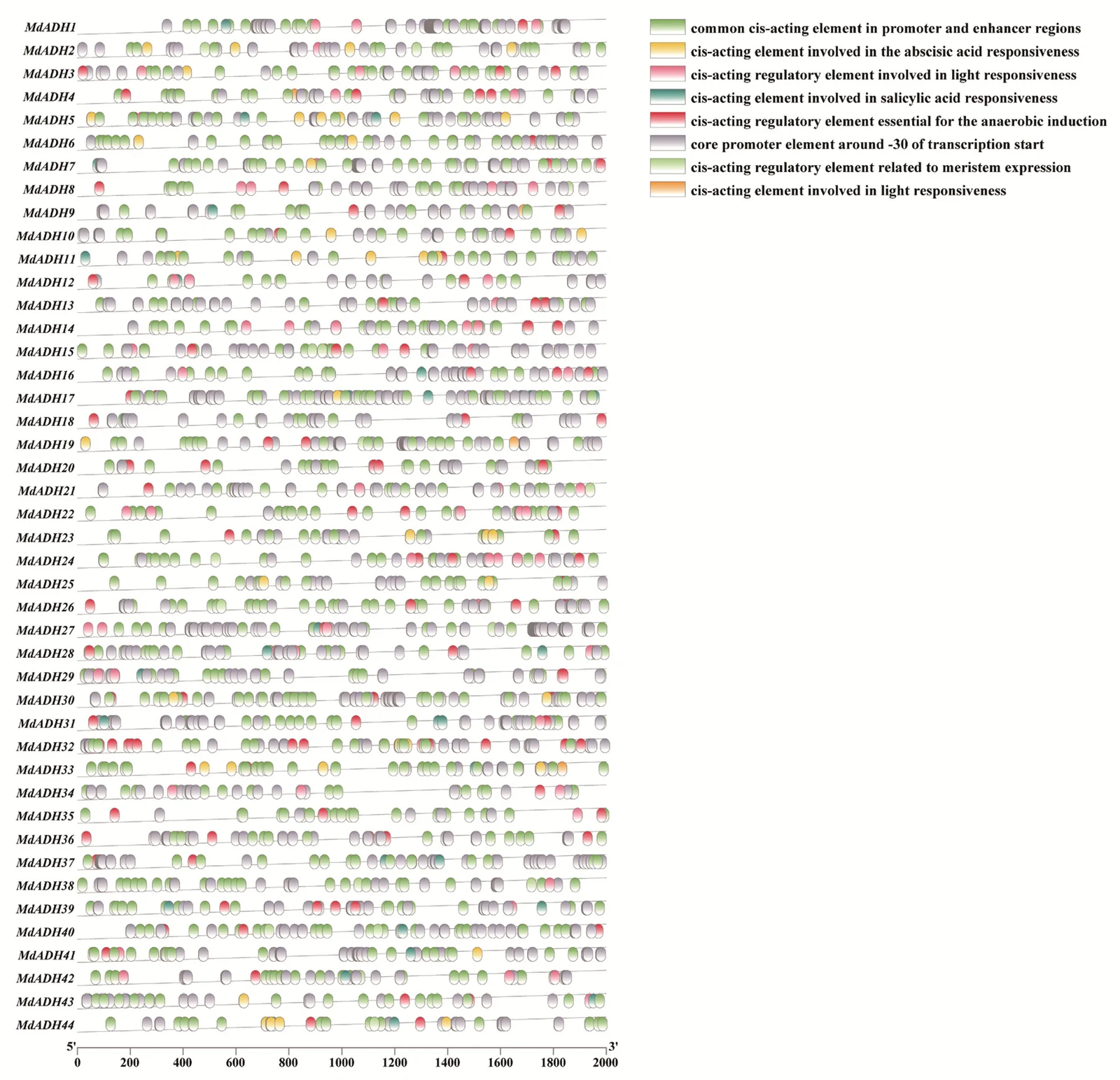
Predicted cis-elements in *MdADH genes* promoters. Cis-elements were predicted using the PlantCARE database based on the 2000 bp upstream sequences of each MdADH gene. Different shapes and colors represent the different types of cis-elements.

To investigate the transcriptional regulation of apple *ADH* genes, we retrieved the 2000 bp upstream sequences of each member and subjected them to cis-element prediction (Figure 4). Eight discrete regulatory motifs were uncovered and classified into four functional categories: development-related, hormone-responsive, stress-responsive, and light-responsive elements. Among these, growth- and development-associated motifs—specifically CAAT-box, TATA-box, and CAT-box—were universally distributed across all *ADH* promoters and constituted the most abundant category. Regarding hormone-responsive elements, ABRE and TCA-element emerged as the dominant types. For stress-responsive motifs, ARE was the major component, present in all *ADH* promoters except that of *MdADH2*, with *MdADH32* containing the highest copy number. Light-responsive elements were predominantly represented by ACE and G-box. Notably, the composition and abundance of these cis-regulatory elements exhibited substantial variation across different ADH family members. *MdADH32*, which encodes the protein with the highest CAI value (Table S1), also harbored the highest combined copy number of stress-related elements among all promoters, followed by *MdADH20* (9 copies) and *MdADH33* (8 copies). In contrast, the promoter of *MdADH28*—the shortest protein (107 aa) and the only mitochondrial-targeted member—contained only four cis-elements in total (two TATA-box, one CAAT-box, and one G-box), lacking any detectable ABRE, ARE, or TCA-element. This sparse promoter architecture suggests that *MdADH28* may be constitutively and minimally expressed, consistent with its potential role as a degenerate or neofunctionalized copy.

### 3.6. Codon Preference Analysis

**Figure 5:**
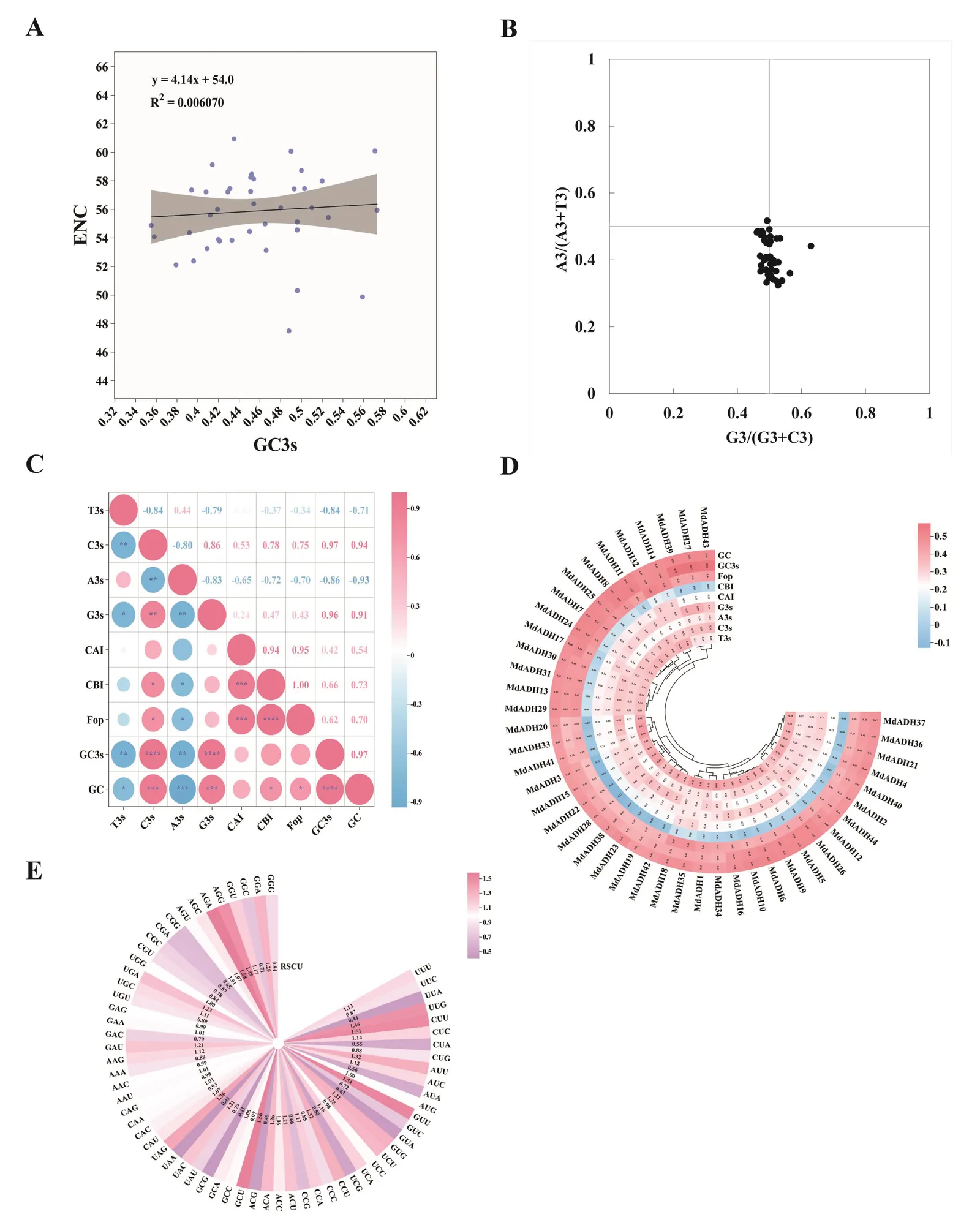
Evolutionary selection pressure and codon usage bias analysis of the MdADH gene family in apple. Codon usage parameters including ENC, CAI, CBI, and RSCU were calculated using CodonW v1.4.2. (**A**) Relative synonymous codon usage (RSCU) of *MdADH* genes. The black solid line denotes the expected neutral evolution curve, and the grey shaded area indicates its 95% confidence interval. (**B**) Correlation analysis of synonymous codon usage parameters, including nucleotide frequencies at the third codon position (A3s, C3s, G3s, T3s, and U3s), codon adaptation index (CAI), codon bias index (CBI), frequency of optimal codons (FOP), and GC content at the third codon position of synonymous codons (GC3s). (**C**) Parity rule 2 (PR2) plot analysis of codon usage bias. The *x*-axis represents G3/(G3 + C3), and the *y*-axis represents A3/(A3 + T3). Deviations from the center point (0.5, 0.5) indicate mutational or selectional constraints shaping codon usage bias. (**D**) ENC-GC3s plot of *MdADH* genes. The *x*-axis represents GC3s, and the *y*-axis represents the effective number of codons (ENC). The solid curve indicates the expected ENC under neutral evolution; genes located below the curve are considered to be shaped by natural selection. Asterisks indicate the significance of differences in parameters between groups: * *p* < 0.05, ** *p* < 0.01, *** *p* < 0.001, **** *p* < 0.0001. (**E**) Correlation analysis of codon usage parameters. Red, blue, and white indicate positive correlation, negative correlation, and no correlation, respectively.

In the apple ADH gene family, 35 frequently used codons with RSCU values greater than 1 were identified through analysis. Among these, the five most frequently used codons were AGA (Arg), GCU (Ala), GUU (Val), and CUU (Leu), with RSCU values ranging from 1.460 to 1.580 (Figure 5A). Furthermore, codon content analysis revealed that the GC, C3s, and FOP. contents of *ADH* genes were concentrated above 0.4, while CBI content was the lowest (Figure 5B). The contents of CAI, G3s, A3s, C3s, and T3s were moderate. CAI is commonly used to predict gene expression levels based on codon sequences, with values ranging between 0 and 1. Evaluation of ENC across the ADH gene family revealed that the majority of members exhibited ENC values falling within the 55–60 interval. Meanwhile, PR2 plot analysis indicated an imbalanced utilization of pyrimidines (T/C) versus purines (A/G) at the third codon position (Figure 5C). Correlation analysis between ENC and GC3s showed that most data points were located near the standard curve (Figure 5D). Codon correlation analysis indicates that GC, GC3s, FOP, and CBI show a positive correlation with C3s, G3s, and CAI, while exhibiting a negative correlation with T3s and A3s (Figure 5E).

### 3.7. Collinearity Analysis

**Figure 6:**
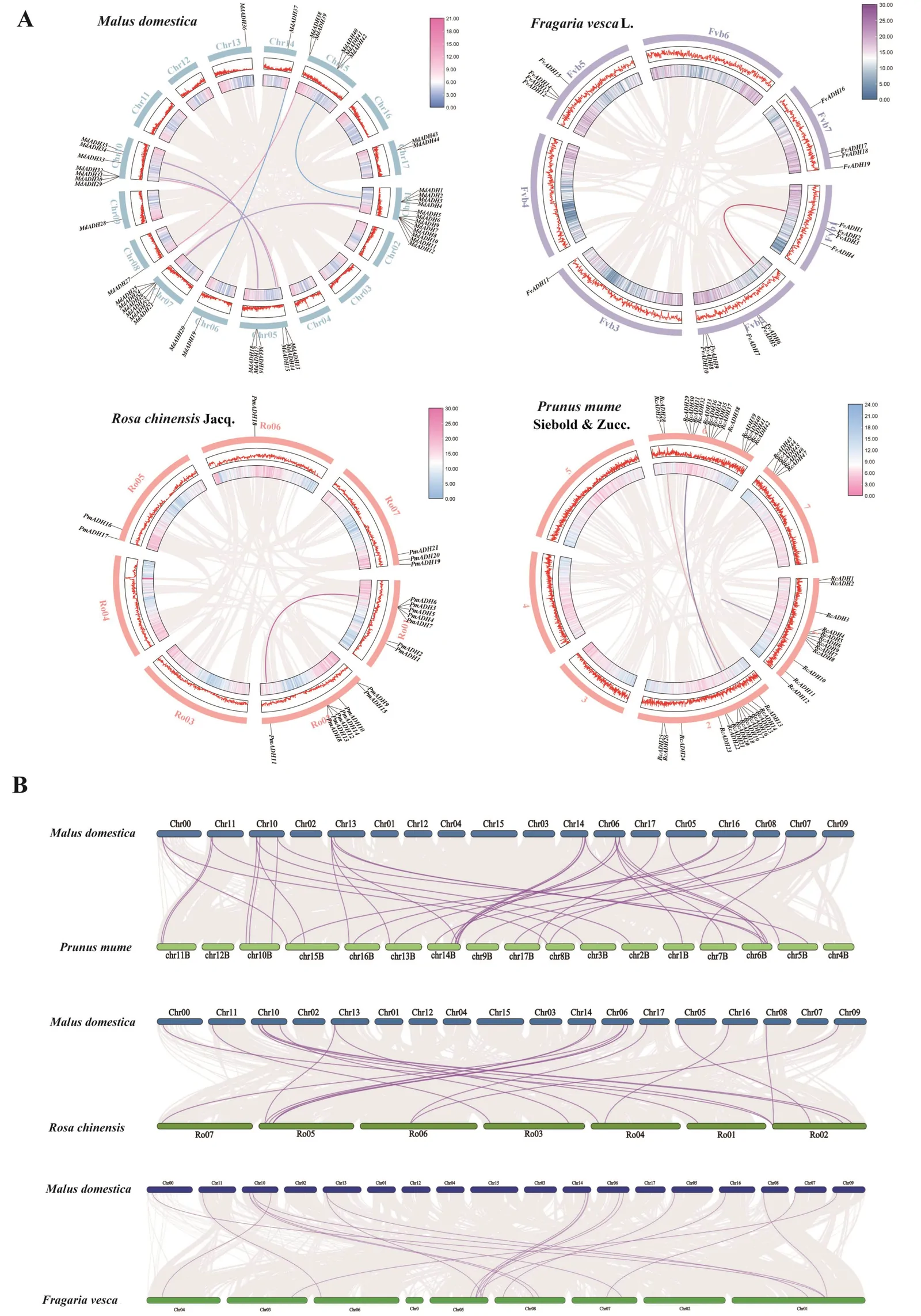
Collinearity relationship analysis of the MdADH gene family in apple. The collinearity analysis was performed using MCScanX with default parameters, and the syntenic blocks were visualized using TBtools-II. (**A**) Intraspecific collinearity relationships among MdADH family genes in the apple genome. In the circular diagram, collinearity gene pairs between *MdADH* genes are connected by colored lines. The outermost and second outermost tracks represent gene density distribution across chromosomes. The gray lines in the background denote syntenic gene pairs within the apple genome. (**B**) Interspecific collinearity relationships of *MdADH* genes between apple and three representative plant species. The purple lines highlight the collinear gene pairs between apple and the corresponding species.

We explored the evolutionary history of apple *ADH* genes through collinearity analyses performed at both the intraspecific and interspecific levels. Intraspecific scrutiny uncovered eight duplicated *ADH* gene pairs within the apple genome, which were largely confined to chromosomes Chr01, Chr05, Chr07, and Chr15 (Figure 6A). At the same time, we conducted a collinearity analysis of plums (Figure 6A), wild strawberries, and roses. To place these genes in a broader phylogenetic context, we built interspecific collinearity maps comparing apple with three other rosaceous species—plum, wild strawberry, and rose (Figure 6B). The resulting data revealed that apple and plum shared the most extensive syntenic relationship, comprising 36 collinear pairs, whereas the numbers of collinear pairs between apple and the other two species were appreciably smaller. Among the 36 syntenic gene pairs shared between apple and plum, 18 pairs (50%) involved apple Chr01, while Chr05 and Chr07 contributed six and five pairs, respectively. By contrast, the syntenic relationships between apple and wild strawberry (11 pairs) and between apple and rose (9 pairs) were not only fewer in number but also distributed across multiple apple chromosomes without prominent hotspots (Chr01 contributing only 3 and 2 pairs, respectively). This differential syntenic density suggests that the Chr01 *ADH* cluster has been preferentially conserved between apple and plum.

### 3.8. Analysis of Tissue-Specific Expression

**Figure 7:**
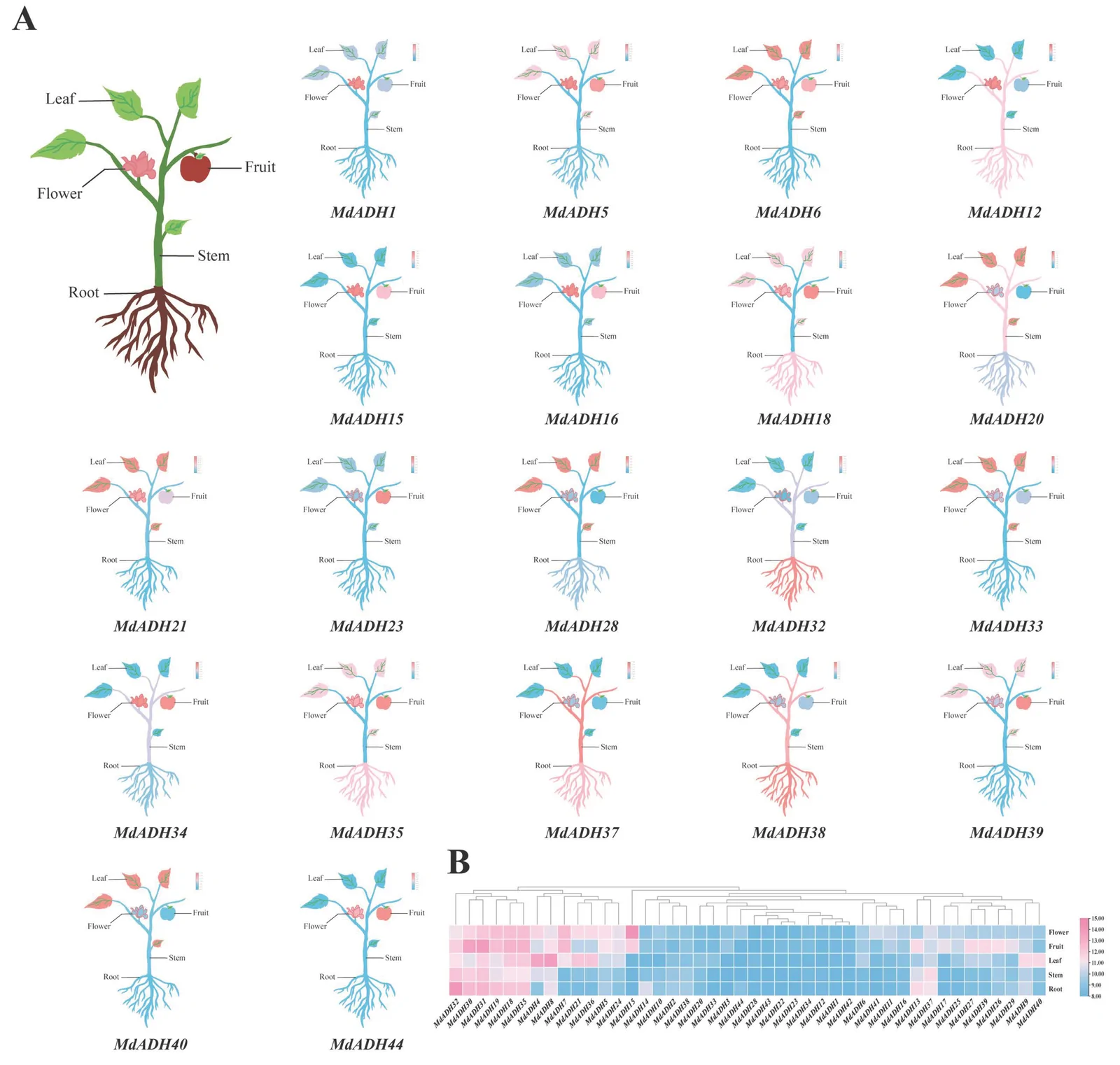
Expression profiles of *MdADH* genes across different apple organs. Expression data (FPKM values) were retrieved from the AppleMDO database. (**A**) Expression levels of *MdADH* genes in various apple organs. (**B**) Heatmap showing the expression patterns of 20 *MdADH* genes. Pink and blue indicate up-regulated and down-regulated expression levels, respectively.

We queried the AppleMDO database for transcriptomic data to evaluate the expression patterns of apple *ADH* genes in five different organs: root, stem, leaf, flower, and fruit (Figure 7). Our findings revealed marked variations in transcript abundances among different *ADH* members within a given tissue, as well as pronounced differences in the expression of any particular gene across the five tissue types. We observed that most *ADH* genes had low expression in roots, although *MdADH32* and *MdADH38* stood out as highly expressed exceptions in this tissue. *MdADH37* was strongly transcribed in roots and stems, but its expression dropped sharply in leaves, flowers, and fruits. Meanwhile, several genes—*MdADH15*, *MdADH20*, and *MdADH33*—showed robust expression throughout multiple organs. In contrast, several genes including *MdADH5*, *MdADH8*, *MdADH23*, *MdADH34*, *MdADH35*, and *MdADH39* exhibited high transcript levels in fruit, suggesting their potential roles in aroma volatile biosynthesis. Notably, several genes from the Chr01 cluster, including *MdADH1* and *MdADH5*, exhibited fruit-specific expression patterns, suggesting a potential functional specialization of this tandem array.

### 3.9. Prediction of Protein Interaction Networks

**Figure 8:**
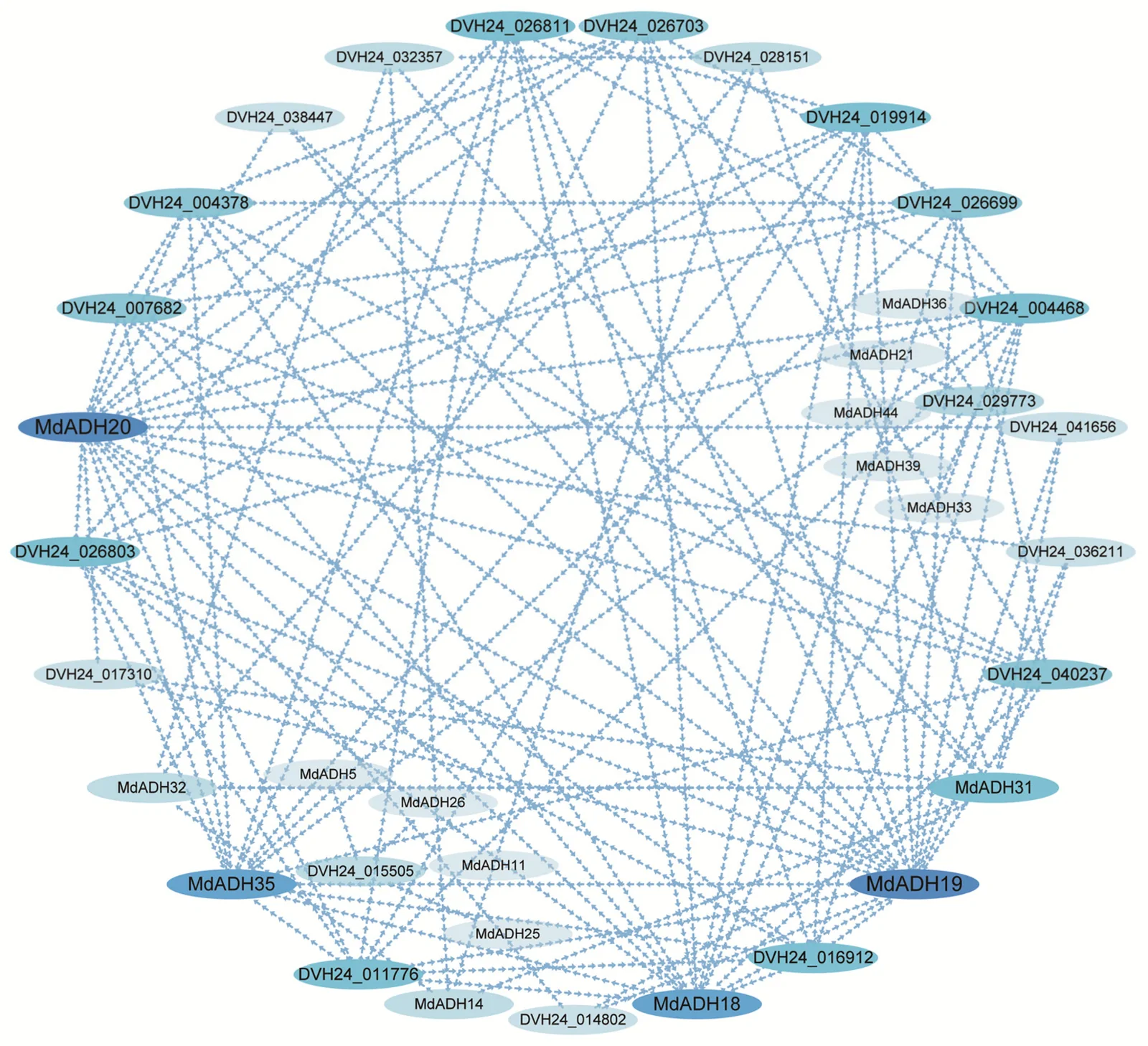
Interaction network among MdADH proteins. Protein–protein interactions were predicted using the STRING database with a minimum confidence score of 0.900 and visualized using Cytoscape v3.9.1. The shades of the ellipses range from dark to light, indicating that the interactions between this protein and other proteins range from significant to insignificant.

To elucidate potential interactions among apple ADH proteins, we employed the STRING database to predict their protein–protein interaction networks. The results revealed that proteins encoded by a total of 16 *ADH* genes were capable of forming an interconnected network (Figure 8). These 16 interacting genes are likely to act as key regulators of ADH family functionality, being closely associated with the biosynthesis of volatile aroma compounds in apple fruit, and may also participate in coordinating growth and developmental processes. Among them, *MdADH20* and *MdADH19* exhibited relatively strong interaction links with other members of the ADH family. The proteins that co-associate with these 16 apple *ADH* genes in the predicted network included, for example, DVH24_026803, DVH24_026811, DVH24_026703, DVH24_004468, and DVH24_007682. Furthermore, these interacting partners could be classified into four functional categories: transcriptional regulation, signal transduction, metabolic modification, and maintenance of protein homeostasis.

### 3.10. Verification of Candidate Gene Transcript Levels in Apple Cultivars by RT-qPCR

**Figure 9:**
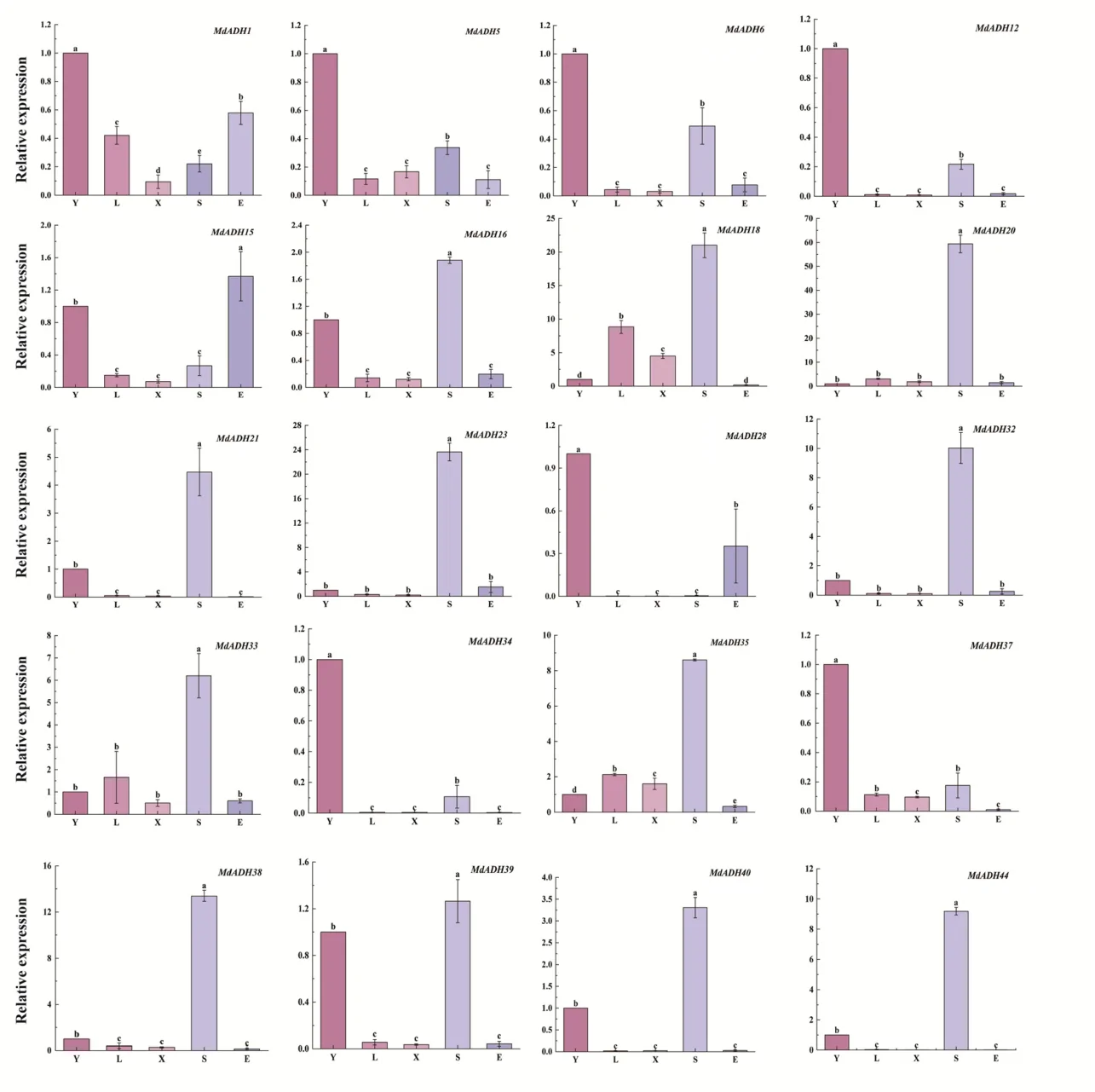
Expression levels of 20 *ADH* genes across different varieties. Statistical analysis was performed using one-way analysis of variance (ANOVA) and Duncan’s multiple range test. The expression level of the Actin gene was set to 1. Black error bars represent the mean ± standard error (SE) of three biological replicates. Different letters indicate significant differences, while the same letters indicate no statistically significant differences (*p* < 0.05). Y, L, X, S, and E stand for ‘Red Delicious’, ‘Starking Red’, ‘Starkrimson’, ‘Red Chief’ and ‘Oregon Spur II’ respectively.

We performed RT-qPCR analysis to verify the transcript levels of selected *ADH* genes in five apple varieties (Figure 9). Among these, the S group showed the highest overall expression (*p* < 0.05). Thirteen genes were significantly induced in S, including *MdADH5*, *MdADH16*, *MdADH18*, *MdADH20*, *MdADH21*, *MdADH23*, *MdADH32*, *MdADH33*, *MdADH35*, *MdADH38*, *MdADH39*, *MdADH40*, and *MdADH44*; of these, *MdADH18*, *MdADH20*, *MdADH23*, *MdADH38*, *MdADH44*, *MdADH35*, and *MdADH33* showed the strongest upregulation. In the E cultivar, *MdADH15* was the most highly expressed gene, with levels significantly above those in other varieties (*p* < 0.05). We also found that *MdADH1*, *MdADH28*, and *MdADH34* were specifically upregulated in E, with their expression in this cultivar significantly exceeding that in all mutant genotypes (*p* < 0.05). The remaining genes did not exhibit any significant expression differences across the five varieties (*p* > 0.05).

## 4. Discussion

### 4.1. Expansion and Evolutionary Drivers of the ADH Gene Family

The apple genome harbors 44 *ADH* genes (Figure 1), a substantial increase in copy number relative to Arabidopsis (8 genes) and tomato (11 genes) [4,17]. This remarkable expansion can be largely ascribed to a tandem duplication event on chromosome Chr01, where a dense cluster of 12 *ADH* genes is situated (Figure 1). Such a finding aligns with the well-documented whole-genome duplication events that have shaped the apple genome [24,25]. In plants, tandem and segmental duplications serve as the principal drivers of gene family enlargement [48], providing raw genetic material for functional divergence and the acquisition of novel functions through copy number proliferation.

Codon preference analysis revealed that *MdADH* gene members exhibit strong preferences for codons such as AGA, GCU, GUU, and CUU (Figure 5A,B); these high-frequency codons can be considered potential optimal codons for the apple ADH gene family. Particularly in heterologous expression systems, codon optimization strategies can significantly enhance the translation efficiency of target genes [49], providing a reference for subsequent genetic engineering applications. Furthermore, *MdADH32* exhibited the highest CAI and CBI values (Figure 5E), suggesting it may possess high translational efficiency and is one of the candidate genes for subsequent protein functional studies. The identification of preferred codons (e.g., AGA, GCU) provides a basis for codon optimization strategies, which could potentially enhance the translational efficiency of apple *ADH* genes in bacterial or yeast expression systems for future functional characterization [32].

Phylogenetic analysis indicates that apple *ADH* genes are closely related to wild strawberry (Figure 2), consistent with their taxonomic status—both belong to the Rosaceae family but are classified under the Maloideae and Rosidoideae subfamilies, respectively [18]. However, interspecies synteny analysis revealed 36 pairs of syntenic gene pairs between apple and plum (Figure 6B), suggesting high homology at the genomic block level [50]. This discrepancy may be related to the complex evolutionary history of the ADH gene family: multiple ancient homologous copies of this family already existed in the Rosaceae ancestor. During subsequent speciation, apple and plum differentially retained different copies of the same ancestral gene, while the strawberry lineage may have undergone independent loss events, leading to inconsistencies between the “gene tree” and the “species tree.” The stronger syntenic relationship observed between apple and plum, compared to strawberry, may reflect differential rates of gene loss after speciation or lineage-specific expansion within the Maloideae subfamily. This highlights the complex evolutionary dynamics of the ADH family, which cannot be fully resolved by phylogenetic analysis alone. Similarly, studies of *ADH* genes in pears and apples have also revealed complex structures of orthology and paralogy; some homologous genes may have become pseudogenes due to loss of function and have undergone processes of neofunctionalization or subfunctionalization [22,23]. Among the 44 *ADH* genes identified in this study, some members have relatively short amino acid sequences (e.g., MdADH28 is only 107 aa) (Table 1).

### 4.2. Protein Physicochemical Features and Subcellular Distribution

Analysis of the physicochemical properties of apple ADH proteins reveals that they generally exhibit high stability (only 2.08% of the proteins had an instability coefficient >40) (Table 1). This high stability may be related to the functional requirements of their role in basal metabolism—as key enzymes in alcohol metabolism [4], they need to maintain relatively stable protein levels to meet the cell’s continuous demand for alcohols. Subcellular distribution prediction indicated that approximately 70% of the ADH members are inferred to localize in the cytoplasm (Table 1), while the remainder, putatively targeted to chloroplasts or other organelles, are speculated to assume more specialized functions, potentially including stress-responsive roles. Previous studies have also shown that ADH enzymes are extensively involved in alcohol synthesis driven by substrate availability and play a role in abiotic stress responses; their high stability facilitates the continuous maintenance of basal metabolism [4,51].

### 4.3. Promoter Cis-Regulatory Element Features

Analysis of promoter cis-element analysis provides important clues for understanding the expression regulation patterns of *ADH* genes (Figure 4). With the exception of *MdADH2*, the promoters of all *ADH* genes contain the ARE cis-regulatory element (Figure 4), which is closely associated with plant adaptation to anaerobic environments. Notably, under anaerobic or waterlogged conditions, ARE activates the expression of downstream genes to adapt to anaerobic environments, and *ADH* genes are among the downstream genes activated by ARE [51]. Based on this, it can be inferred that these genes participate in plant adaptation to anaerobic environments. This study also found that growth and development-related regulatory elements were the most abundant (Figure 4), which is consistent with their involvement in physiological functions such as fruit development, ripening, and stress responses [52]. Additionally, tissue-specific analysis results showed that many genes exhibited high expression levels in fruit, which aligns with the role of *ADH* genes in regulating aroma compounds in apple fruit [3,27]. Furthermore, this study found that most apple *ADH* genes exhibit higher expression levels in mature fruit compared to other organs (Figure 7), and the majority of *ADH* genes contain ABRE cis-regulatory elements (involved in the response to abscisic acid signaling), suggesting that their transcription may be regulated by abscisic acid [53]; however, this hypothesis requires experimental validation. The variation in both the categories and copy numbers of cis-elements among different genes suggests that individual *ADH* members may perceive and respond to distinct endogenous and environmental cues. Nevertheless, the apple ADH gene family as a whole exhibits a clear trend of correlation with growth and development. Therefore, it is speculated that the elements within this family are both conserved and exhibit differentiation across different genes during development. Unlike the *HPL* genes—which are also part of the LOX pathway and are characterized by a high abundance of light-responsive and ABA-responsive elements—*ADH* genes are more focused on responding to growth, development, and endogenous signals [54,55]. This differentiation in regulatory elements may lead to distinct expression patterns of *ADH* and *HPL* genes during different stages of fruit development or under varying environmental conditions, resulting in a division of labor and synergistic coordination between the two in the aroma synthesis pathway. More over, The positive correlation between the CAI value and the abundance of stress-responsive cis-elements in the promoter of *MdADH32* suggests that both translational efficiency and transcriptional regulation might have co-evolved to ensure rapid and abundant protein production under specific stress conditions.

### 4.4. Analysis of Protein Interaction Networks and Tissue Expression Patterns

In the predicted protein interaction network (Figure 8), MdADH19 and MdADH20 are suggested to occupy central positions, with potential interactions predicted for several proteins, including DVH24_026803 (NAD(P)-binding domain superfamily), DVH24_026811 (NAD(P)-binding domain superfamily), DVH24_026703 (NAD(P)-dependent oxidoreductase domain superfamily) [55,56], DVH24_004468 (NAD(P)-binding domain superfamily), and DVH24_007682 (inositol oxidase) [57], among others. Based on their predicted domain compositions, these interactors can be tentatively assigned to four functional categories: transcriptional regulation, signal transduction, metabolic modification, and protein homeostasis maintenance, and they might potentially coordinate enzyme complex assembly and metabolic flux allocation. Among them, DVH24_026703 is noteworthy for containing an Aldo_ket_red domain. The NAD(P)-dependent oxidoreductase domain superfamily is known to include proteins with Aldo_ket_red domains, and members of this superfamily are generally inferred to catalyze redox reactions using NADPH as a substrate [55,56]; similarly, NAD(P)H dehydrogenase (NDH) complexes are reported to play a key role in cyclic electron transport during photosynthesis and respiration, while also promoting ATP formation [58,59]. Among these predicted interactors, DVH24_026703 is noteworthy for harboring an Aldo_ket_red domain, a signature conserved domain of the NAD(P)-dependent oxidoreductase superfamily (IPR036812) [56]. Members of this superfamily are characterized by their ability to catalyze redox reactions using NAD(P)H as a cofactor and are often implicated in secondary metabolism and stress responses in plants [59]. Given that ADH enzymes also function as NAD(P)-dependent oxidoreductases in the conversion of aldehydes to alcohols, the predicted interaction between MdADH proteins and DVH24_026703, both sharing oxidoreductase activity, raises the possibility that they may functionally cooperate in modulating the redox balance or metabolic flux within the aroma biosynthetic pathway [59]. However, it should be noted that these predictions are solely derived from computational analysis. Their biological relevance and whether they indeed interact with MdADH proteins in the context of fruit aroma biosynthesis require experimental validation, such as yeast two-hybrid assays and enzymatic activity characterization. Although these proteins are primarily annotated as photosynthetic based on current databases, whether they are actually expressed in fruit organs and whether they cooperate with *ADH* genes to influence the production or supply of volatile precursors remain open questions that require experimental validation. We also observed marked organ-dependent expression differences among apple *ADH* genes (Figure 7), which may suggest functional specialization during the evolution of this family. Such divergence could reflect either subfunctionalization of the ancestral gene’s original roles among different members, or neofunctionalization of particular copies.

### 4.5. Screening and Functional Prediction of Core Candidate Genes

RT-qPCR analysis results showed that different genes exhibited diverse expression patterns (Figure 9). A small number of genes displayed distinct expression preferences. *MdADH15* was most highly expressed in the E cultivar (1.37), significantly higher than in the Y cultivar (1.00) and other mutated varieties (0.07–0.27). *MdADH28* was expressed at higher levels in the Y (1.00) and E (0.35) varieties than in L, X, and S (all below 0.01). *MdADH1* and *MdADH34* were highly expressed primarily in the Y cultivar, with significantly lower expression levels in the other varieties. In contrast, a large number of genes showed significant variety-specific upregulation in the S cultivar (Figure 9), suggesting a coordinated transcriptional response in this cultivar. Among these, *MdADH20* exhibited the highest relative expression level (59.38) in the S cultivar, making it a core candidate gene for significant variety-specific upregulation in the S cultivar. The pronounced upregulation of *MdADH20* in the S cultivar suggests a correlative association with enhanced ester biosynthesis potential. However, direct functional evidence including enzymatic activity assays and genetic transformation is required to confirm its causative role in apple aroma formation. Nevertheless, its expression pattern and central position in the interaction network render it a high-priority candidate for future mechanistic studies. Notably, MdADH28 is the member with the shortest amino acid sequence (Table 1), yet it is expressed in multiple tissues (Figure 7), suggesting that it may be a highly specialized and efficient core enzyme or a truncated form with a specific regulatory function. Previous studies on apple aroma compounds have found that genes specifically expressed in apples are positively correlated with aroma compounds such as ethyl hexanoate [26,60,61,62,63,64]; the relationship between these genes and the core candidate genes identified in this study warrants further investigation [65].

### 4.6. Study Limitations and Perspectives

While our bioinformatics-based investigation has provided a thorough overview of the apple ADH gene family, some unresolved issues persist [3,59,66]. First, the specific biochemical functions of the identified key candidate genes (such as *MdADH20*) in aroma synthesis have not yet been validated through in vitro enzyme activity assays or in vivo genetic transformation. Second, the regulatory relationships between the predicted cis-acting elements in the promoters and specific cis-element analysis require experimental confirmation (Figure 4). In addition, a more comprehensive integrative analysis is required to establish the association between gene transcript levels and the accumulation dynamics of specific aroma volatiles, both among different apple varieties and across various fruit maturation stages. Future research should integrate metabolomics analysis to establish a network linking gene expression and the accumulation of aroma compounds; utilize transient expression systems or stable genetic transformation systems to validate the functions of key genes; elucidate the interaction partners and complex composition of the hub proteins MdADH19/20; and conduct genetic engineering application studies based on the results of codon preference analysis (Figure 5). A major limitation of this study is the absence of in vitro biochemical assays to confirm the enzymatic activities and substrate specificities of the identified ADH proteins. Additionally, the promoter activities of key genes like *MdADH20* and the effect of codon optimization on their heterologous expression warrant further exploration.

## 5. Conclusions

**Figure 10:**
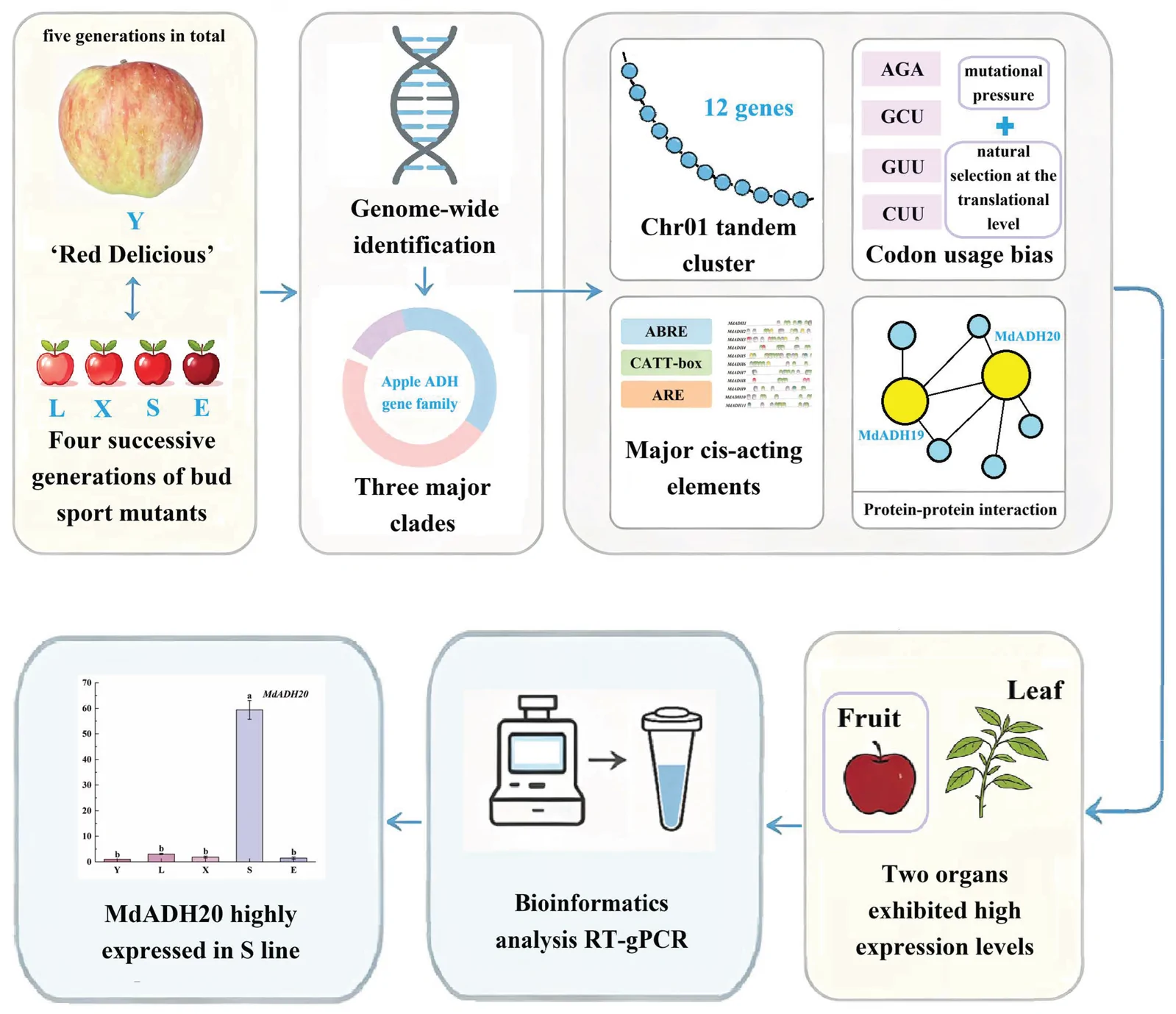
Panel-based workflow summarizing the identification and expression characterization of the MdADH gene family in ‘Red Delicious’ apple and its four successive generations of bud-sport mutants. Starting with apple germplasm materials, genome-wide screening classified the apple ADH family members into three major clades. Multiple bioinformatic analyses were subsequently performed, including identification of the tandem-duplicated gene cluster on the chromosome, evaluation of codon-usage bias, prediction of promoter cis-regulatory elements, and protein-protein interaction prediction. Finally, tissue-specific expression analysis and RT-qPCR detection were conducted.

In conclusion, this work provides the first genome-wide survey of the ADH gene family within the context of the LOX-derived aroma biosynthetic pathway in apple (Figure 10). Our investigation uncovered 44 *ADH* members, among which 12 are arranged as a tight cluster on chromosome Chr01. These findings suggest that the family has undergone expansion primarily via tandem duplications during the course of evolution, while also displaying both conserved features and lineage-specific divergence among Rosaceae species. The encoded proteins are generally highly stable and predominantly cytoplasmic in localization, a pattern consistent with their housekeeping roles in primary metabolism. Phylogenetic analysis indicates that apple is evolutionarily closely related to wild strawberry, whereas linkage analysis reveals a higher degree of linkage with plum. The promoters of apple *ADH* genes are rich in elements associated with growth and development, and codon preference analysis identified potential optimal codons such as AGA, GCU, GUU, and CUU. Protein interaction network predictions identified *MdADH19* and *MdADH20* as hub genes within the family. Tissue expression profiling and RT-qPCR analyses of four-generation mutants of ‘Red Delicious’ ripe fruit screened out highly expressed core candidate genes such as *MdADH20*. This work established a comprehensive framework for the apple ADH gene family, enhanced our knowledge of the molecular basis governing fruit aroma biosynthesis [67], and furnished a set of candidate gene targets that may facilitate aroma-oriented screening in bud mutation breeding programs. It should be emphasized that the candidate genes prioritized in this study, including *MdADH20*, are identified through integrative genomic and transcriptomic screening; their proposed functions in aroma biosynthesis await experimental validation through biochemical and genetic approaches in future investigations. It also offers important genetic resources and theoretical references for improving apple flavor quality through biotechnological approaches.

## Figures and Tables

**Table 1:** Physicochemical properties and subcellular localization of the 44 ADH proteins identified in apple (*Malus domestica*).

Gene ID	Gene Name	Number of Amino Acid	Molecular Weight	Theoretical PI	Instability Index	Aliphatic Index	Grand Average of Hydropathicity	Subcellular Localization
MD01G1041700	*MdADH1*	363	39,297.53	6.17	30.92	93.99	0.074	Cytoskeleton
MD01G1042500	*MdADH2*	361	39,072.30	6.06	23.32	91.75	0.095	Cytoplasm
MD01G1042900	*MdADH3*	361	39,202.38	6.31	26.25	93.88	0.051	Cytoplasm
MD01G1043100	*MdADH4*	362	38,893.84	6.63	26.99	89.92	−0.004	Cytoplasm
MD01G1110100	*MdADH5*	371	40,171.61	6.47	26.02	94.29	0.015	Cytoplasm
MD01G1194600	*MdADH6*	358	38,377.75	6.75	29.11	90.92	0.111	Extracellular
MD01G1194700	*MdADH7*	368	39,486.24	7.07	21.56	91.60	0.110	Extracellular
MD01G1195200	*MdADH8*	368	39,313.87	7.51	24.96	90.00	0.098	Extracellular
MD01G1195300	*MdADH9*	368	39,534.24	7.50	25.79	89.76	0.077	Cytoplasm
MD01G1195400	*MdADH10*	368	39,444.87	6.27	25.22	89.21	0.093	Cytoplasm
MD01G1195500	*MdADH11*	365	38,884.18	7.51	20.33	88.41	0.042	Cytoplasm
MD01G1238700	*MdADH12*	386	41,401.94	5.96	37.07	93.63	0.061	Cytoplasm
MD05G1013200	*MdADH13*	383	42,019.50	6.86	33.65	85.69	−0.083	Cytoplasm
MD05G1013400	*MdADH14*	379	41,166.15	6.15	34.92	79.95	−0.121	Cytoplasm
MD05G1034100	*MdADH15*	359	38,578.62	6.26	30.07	90.36	0.045	Cytoplasm
MD05G1179800	*MdADH16*	380	41,161.44	5.79	31.57	86.42	0.007	Extracellular
MD05G1180800	*MdADH17*	415	45,415.32	7.94	33.54	84.31	−0.151	Cytoplasm
MD05G1186000	*MdADH18*	379	40,614.86	6.51	25.73	88.18	0.058	Cytoplasm
MD06G1121600	*MdADH19*	396	42,833.15	6.47	21.60	82.42	−0.073	Cytoplasm
MD06G1241000	*MdADH20*	393	42,205.33	5.61	37.79	87.25	0.033	Cytoplasm
MD07G1183600	*MdADH21*	360	39,288.18	6.20	27.97	90.14	−0.079	Cytoplasm
MD07G1250800	*MdADH22*	324	34,647.76	5.94	36.09	97.44	0.060	Cytoplasm
MD07G1251000	*MdADH23*	326	34,812.87	6.01	33.82	96.53	0.045	Cytoplasm
MD07G1261900	*MdADH24*	368	39,466.94	6.46	23.21	91.58	0.085	Cytoplasm
MD07G1262100	*MdADH25*	368	39,704.31	7.05	21.84	90.00	0.039	Cytoplasm
MD07G1262300	*MdADH26*	366	39,142.36	7.52	20.52	88.42	−0.002	Cytoplasm
MD08G1009200	*MdADH27*	370	40,038.90	6.53	25.81	89.30	−0.072	Peroxisomes
MD09G1132700	*MdADH28*	107	12,120.18	9.14	33.10	73.83	−0.208	Mitochondria
MD10G1013800	*MdADH29*	372	40,372.39	5.83	32.59	86.67	−0.028	Cytoplasm
MD10G1013900	*MdADH30*	380	41,432.73	6.68	30.50	87.16	−0.031	Cytoplasm
MD10G1014200	*MdADH31*	380	41,408.62	6.47	30.06	86.16	−0.064	Cytoplasm
MD10G1014500	*MdADH32*	379	41,138.16	6.03	37.88	79.95	−0.095	Cytoplasm
MD10G1097200	*MdADH33*	355	38,529.30	5.83	24.45	88.03	−0.053	Cytoplasm
MD10G1155000	*MdADH34*	359	39,003.25	6.42	31.35	90.08	0.037	Cytoplasm
MD10G1174100	*MdADH35*	379	40,490.63	6.75	26.57	85.86	0.037	Cytoplasm
MD13G1280800	*MdADH36*	358	38,926.84	6.89	29.58	92.82	−0.034	Cytoplasm
MD14G1142100	*MdADH37*	357	38,474.99	6.63	30.82	82.44	−0.056	Cytoplasm
MD15G1004100	*MdADH38*	391	42,522.17	6.04	40.62	90.97	0.057	Chloroplast
MD15G1008100	*MdADH39*	366	39,941.07	8.24	27.45	85.46	−0.120	Peroxisomes
MD15G1308800	*MdADH40*	361	39,110.62	6.39	29.19	95.54	0.107	Cytoplasm
MD15G1308900	*MdADH41*	361	38,858.12	8.46	27.94	91.52	−0.003	Cytoplasm
MD15G1309000	*MdADH42*	363	39,380.56	6.00	31.62	94.74	0.055	Cytoplasm
MD17G1119700	*MdADH43*	358	39,018.94	6.19	30.89	88.44	0.031	Cytoplasm
MD17G1119800	*MdADH44*	357	38,499.12	6.41	32.10	89.52	0.009	Cytoplasm

Notes: Number of amino acid is given in amino acids (aa); molecular weight in kDa; pI, theoretical isoelectric point; Instability index, aliphatic index, and grand average of hydropathicity are dimensionless values. Subcellular localization was predicted using WoLF PSORT.

## Data Availability

The datasets used and analyzed in this study are available upon reasonable request to the corresponding author.

## Supplementary Materials

The following supporting information can be downloaded at: https://www.mdpi.com/article/10.3390/biology15171486/s1, Table S1: Data on codon usage preferences of members of the Apple ADH gene family. Table S2: *ADH* gene expression levels in various plant organs. Table S3: Primer sequences for 20 *ADH* genes for tissue-specific expression profiling and RT-qPCR analysis.
