# Supplementary material for: Molecular Evolution and Expression Analysis of the ADH Gene Family in Apple Bud Mutants
Source: Biology (Basel). 2026 Sep 2;15(17):1486. doi: 10.3390/biology15171486 (PMC13565118; doi:10.3390/biology15171486)
Supplement: Supplementary file 1 [file biology-15-01486-s001.zip › biology-4483062-supplementary.pdf]

Table S1 Data on codon usage preferences of members of the Apple ADH gene family.

Sample	T3s	C3s	A3s	G3s	CAI	CBI	Fop	GC3s	GC	Nc(ENC)	L_sym	L_aa	Gravy	Aromo
MdADH1	0.4089	0.2852	0.3142	0.2469	0.244	0.033	0.443	0.414	0.449	59.13	343	358	0.030726	0.089385
MdADH2	0.4048	0.2789	0.3011	0.2541	0.207	-0.025	0.398	0.419	0.461	56	344	361	0.107479	0.063712
MdADH3	0.3967	0.2667	0.297	0.2851	0.189	-0.111	0.349	0.429	0.454	57.22	347	361	0.05097	0.074792
MdADH4	0.388	0.2943	0.2784	0.2738	0.23	0	0.418	0.45	0.483	54.45	349	362	-0.003591	0.071823
MdADH5	0.3953	0.3074	0.2107	0.3269	0.24	0.002	0.42	0.5	0.505	58.72	352	366	-0.002459	0.051913
MdADH6	0.4048	0.3045	0.2022	0.3227	0.235	-0.012	0.408	0.496	0.51	55.12	341	358	0.110614	0.053073
MdADH7	0.367	0.3367	0.2274	0.3074	0.264	0.097	0.47	0.51	0.51	56.12	351	368	0.110054	0.05163
MdADH8	0.3754	0.3389	0.2007	0.3115	0.261	0.1	0.472	0.52	0.522	57.99	352	368	0.098098	0.05163
MdADH9	0.4	0.2983	0.2143	0.3269	0.231	-0.002	0.413	0.493	0.505	57.43	351	368	0.077446	0.054348
MdADH10	0.4027	0.3054	0.2214	0.2962	0.216	-0.044	0.389	0.48	0.508	56.11	350	368	0.093206	0.054348
MdADH11	0.3593	0.3559	0.2174	0.3074	0.249	0.025	0.434	0.526	0.514	55.43	350	365	0.041644	0.052055
MdADH12	0.3301	0.3141	0.3179	0.2717	0.179	-0.02	0.403	0.465	0.479	54.98	372	386	0.061399	0.056995
MdADH13	0.3555	0.3156	0.3288	0.2618	0.245	0.082	0.468	0.451	0.472	57.25	370	383	-0.082768	0.08094
MdADH14	0.396	0.3234	0.2313	0.3206	0.305	0.126	0.499	0.496	0.491	50.31	367	379	-0.120581	0.076517
MdADH15	0.3967	0.2667	0.297	0.2851	0.189	-0.111	0.349	0.429	0.454	57.22	347	361	0.05097	0.074792
MdADH16	0.4058	0.263	0.2431	0.3384	0.207	-0.002	0.419	0.466	0.479	53.13	365	380	0.006842	0.071053
MdADH17	0.3323	0.3263	0.2945	0.3147	0.233	0.023	0.434	0.496	0.48	54.56	401	415	-0.150843	0.072289
MdADH18	0.4521	0.264	0.2703	0.2574	0.284	0.082	0.471	0.409	0.479	53.24	367	379	0.05752	0.073879
MdADH19	0.4006	0.265	0.3522	0.24	0.202	-0.092	0.373	0.392	0.451	54.37	383	396	-0.073232	0.075758
MdADH20	0.4063	0.2313	0.3795	0.2194	0.204	-0.07	0.382	0.355	0.454	54.88	380	393	0.032825	0.066158
MdADH21	0.3953	0.2591	0.2939	0.3029	0.224	-0.046	0.389	0.435	0.467	60.94	347	360	-0.078611	0.075
MdADH22	0.4286	0.2208	0.3165	0.3067	0.193	-0.127	0.357	0.408	0.462	57.22	98	104	-0.166346	0.125
MdADH23	0.3866	0.2305	0.3358	0.251	0.207	-0.075	0.366	0.394	0.492	57.36	320	326	0.044785	0.064417
MdADH24	0.3612	0.3411	0.2401	0.2874	0.261	0.086	0.463	0.503	0.513	57.45	352	368	0.085326	0.05163

MdADH25	0.3919	0.3176	0.2258	0.3012	0.248	0.084	0.462	0.49	0.505	60.07	351	368	0.038587	0.05163
MdADH26	0.3953	0.3074	0.2107	0.3269	0.24	0.002	0.42	0.5	0.505	58.72	352	366	-0.002459	0.051913
MdADH27	0.276	0.3474	0.2555	0.3944	0.213	0.007	0.427	0.571	0.506	60.09	361	370	-0.071892	0.072973
MdADH28	0.4286	0.2208	0.3165	0.3067	0.193	-0.127	0.357	0.408	0.462	57.22	98	104	-0.166346	0.125
MdADH29	0.348	0.3074	0.3275	0.2704	0.253	0.082	0.468	0.454	0.48	58.13	361	372	-0.028226	0.075269
MdADH30	0.3443	0.3115	0.3356	0.2657	0.24	0.05	0.451	0.451	0.475	58.24	370	380	-0.063947	0.076316
MdADH31	0.3443	0.3148	0.3333	0.2667	0.24	0.056	0.454	0.454	0.472	56.4	370	380	-0.030526	0.078947
MdADH32	0.4073	0.3179	0.2297	0.3168	0.315	0.164	0.52	0.488	0.486	47.49	367	379	-0.095251	0.076517
MdADH33	0.443	0.2282	0.3462	0.2333	0.23	-0.046	0.402	0.358	0.44	54.08	346	357	-0.055742	0.084034
MdADH34	0.4433	0.2367	0.289	0.275	0.234	0.01	0.428	0.396	0.445	52.38	346	357	0.008964	0.078431
MdADH35	0.4492	0.2721	0.2576	0.2591	0.302	0.095	0.48	0.42	0.486	53.89	367	379	0.037467	0.073879
MdADH36	0.3828	0.2706	0.2814	0.3112	0.221	-0.055	0.383	0.452	0.47	58.46	347	358	-0.034358	0.078212
MdADH37	0.3828	0.2706	0.2814	0.3112	0.221	-0.055	0.383	0.452	0.47	58.46	347	358	-0.034358	0.078212
MdADH38	0.3478	0.2671	0.3579	0.2674	0.18	-0.1	0.357	0.421	0.471	53.78	378	391	0.056777	0.071611
MdADH39	0.2718	0.3456	0.2593	0.4016	0.215	0.011	0.429	0.573	0.502	55.95	354	366	-0.120492	0.07377
MdADH40	0.3986	0.2921	0.2955	0.2562	0.2	-0.066	0.378	0.431	0.453	57.45	341	359	0.037047	0.077994
MdADH41	0.4524	0.2449	0.3018	0.239	0.218	-0.046	0.391	0.379	0.447	52.11	348	361	-0.003324	0.066482
MdADH42	0.3844	0.2721	0.3321	0.2531	0.205	-0.065	0.38	0.412	0.456	55.6	345	363	0.073829	0.066116
MdADH43	0.2544	0.3624	0.2895	0.3426	0.222	0.047	0.444	0.559	0.504	49.86	340	355	-0.052958	0.070423
MdADH44	0.3593	0.2881	0.3382	0.2651	0.225	-0.013	0.407	0.433	0.463	53.84	349	363	0.055372	0.068871

Table S2 *ADH* gene expression levels in various plant organs.

Gene ID	Gene	Flower	Fruit	Leaf	Stem	Root
MD01G1041700	*MdADH1*	8.5	8.3	8.3	8.2	8.2
MD01G1042500	*MdADH2*	9.2	8.5	9.0	9.3	9.5
MD01G1042900	*MdADH3*	9.0	9.3	8.0	8.0	8.1

MD01G1043100	*MdADH4*	11.6	10.5	13.4	10.6	9.1
MD01G1110100	*MdADH5*	11.3	11.1	10.4	9.5	9.7
MD01G1194600	*MdADH6*	9.7	9.5	9.9	8.5	8.5
MD01G1194700	*MdADH7*	12.6	12.7	11.0	8.5	8.5
MD01G1195200	*MdADH8*	10.9	11.3	13.8	10.7	10.9
MD01G1195300	*MdADH9*	9.8	10.2	11.4	9.9	10.3
MD01G1195400	*MdADH10*	9.5	9.0	8.2	8.9	9.2
MD01G1195500	*MdADH11*	10.3	9.9	8.6	8.4	8.3
MD01G1238700	*MdADH12*	8.5	8.2	8.1	8.3	8.3
MD05G1013200	*MdADH13*	9.5	11.4	9.6	10.6	11.4
MD05G1013400	*MdADH14*	8.9	9.1	8.3	8.9	10.6
MD05G1034100	*MdADH15*	14.3	11.8	8.2	8.1	8.1
MD05G1179800	*MdADH16*	9.9	9.4	8.9	8.6	8.6
MD05G1180800	*MdADH17*	9.9	10.8	8.6	8.3	8.3
MD05G1186000	*MdADH18*	12.4	12.9	12.0	11.1	12.1
MD06G1121600	*MdADH19*	12.4	12.4	10.9	10.9	12.3
MD06G1241000	*MdADH20*	8.7	8.5	9.1	8.8	8.7
MD07G1183600	*MdADH21*	11.4	10.1	12.1	9.0	8.5
MD07G1250800	*MdADH22*	8.2	8.5	8.2	8.1	8.1
MD07G1251000	*MdADH23*	8.2	8.5	8.2	8.1	8.1
MD07G1261900	*MdADH24*	10.9	10.9	10.5	9.2	9.0
MD07G1262100	*MdADH25*	10.1	10.3	9.4	8.3	8.3
MD07G1262300	*MdADH26*	9.8	11.3	9.5	9.5	9.3
MD08G1009200	*MdADH27*	9.7	11.5	10.1	8.7	8.7
MD09G1132700	*MdADH28*	8.2	8.1	8.5	8.1	8.2
MD10G1013800	*MdADH29*	9.7	10.9	9.1	9.0	9.5

MD10G1013900	*MdADH30*	11.6	13.6	10.8	11.8	12.6
MD10G1014200	*MdADH31*	12.1	13.9	11.7	12.1	12.8
MD10G1014500	*MdADH32*	11.0	11.8	11.2	12.2	14.1
MD10G1097200	*MdADH33*	8.5	8.5	9.1	8.2	8.2
MD10G1155000	*MdADH34*	8.7	8.7	8.2	8.4	8.3
MD10G1174100	*MdADH35*	12.4	12.9	12.0	11.1	12.1
MD13G1280800	*MdADH36*	11.4	10.1	12.1	9.0	8.5
MD14G1142100	*MdADH37*	10.6	10.4	10.3	11.4	11.0
MD15G1004100	*MdADH38*	9.1	9.1	8.9	9.4	9.6
MD15G1008100	*MdADH39*	9.7	11.5	10.1	8.7	8.7
MD15G1308800	*MdADH40*	9.4	9.0	11.5	9.1	8.8
MD15G1308900	*MdADH41*	10.4	9.0	8.5	8.1	8.1
MD15G1309000	*MdADH42*	8.5	8.5	8.3	8.2	8.2
MD17G1119700	*MdADH43*	8.3	8.2	8.1	8.0	8.0
MD17G1119800	*MdADH44*	9.3	8.0	8.0	8.0	8.9

Table S3. Primer sequences for 20 *ADH* genes for tissue-specific expression profiling and RT-qPCR analysis

Gene ID	Forward (5’ to 3’)	Forward (5’ to 3’)
MD01G1041700	GCCATGGCTAAATCCCTCGA	AACCATGACAAAAACGCCGG
MD01G1110100	TCACTTCTGCTTCTGCCAGG	ACGAGCACTGGTCTCAAAGG
MD01G1194600	TGTGCTTTAAGCTCCCGGAG	GGCTCCGATGATCAGGACAG
MD01G1238700	GAAGCGCTTGAAGCCACAAA	GGAAGGTCGGTCTTCGGTTT
MD05G1034100	ATCATGGTGGCCAATGAGCA	TACCGGGTTTGTCAAGTCCG
MD05G1179800	GGTGGAGCTGACAGAAGCAT	CCACACCCCAACCATCATGA
MD05G1186000	CAGCAAAGGTAGAAGCGGGA	CCAGCTGTTTTGGCACCTTC

MD06G1241000	CTGTGGTGCACTCAGGATGT	CAACATTCCAAGCTGCACCC
MD07G1183600	CGGAAAGCATTTGGGTGTGG	TGCCTCATCCTCCTTTGCTG
MD07G1251000	ATGAACTTGGCGGTCCTGAG	TAGGGAAGCGTAGGAGCCTT
MD09G1132700	TTGCTCCACCTAAGGCCATG	ACCTTCAGTAACAGCGAGGC
MD10G1014500	GGACAACAACCGCTGTTTCC	CCAGTGAACACAGGGAGGAC
MD10G1097200	GCGTTGGATATGCTGCAAGG	AACATCGTCAGCCCCAAGAG
MD10G1155000	AAGAGGAAATGACGGCCCTG	CACCAGCAGTCTTCAGCAGA
MD10G1174100	TGCCTTCTTGGTTGTGGTGT	CCAGCTGTTTTGGCACCTTC
MD14G1142100	AGAGACTCGGGGCTGATTCT	TGGAGCCAGAGCATGAACTG
MD15G1004100	CGGAGGGAGCAAGACTTTGT	AAGCAATAGTCTGCACCCCC
MD15G1008100	GACGGCAGTATCACCTACGG	TGCATCCAACGGCAGATTCT
MD15G1308800	ATCATGGTGGCCAATGAGCA	CACCCACATGCATACCTGGT
MD15G1308900	ATTGCAGGTTGCCATGGGTA	GATTGGAGCACCCACCAGAA
MdActin	TGACCGAATGAGCAAGGAAATTACT	TACTCAGCTTTGGCAATCCACATC
